# Supplementary material for: From sequence to enzyme mechanism using multi-label machine learning
Source: BMC Bioinformatics. 2014 May 19;15:150. doi: 10.1186/1471-2105-15-150 (PMC4229970; doi:10.1186/1471-2105-15-150)
Supplement: Additional file 2 — Java code of ml2db. Additional file ml2db_code.tar.gz contains the Java source code to run the multi-label machine learning experiments and save the results to database. The code’s Javadoc is included. [file 1471-2105-15-150-S2.zip › additional file 2/ml2db/utils/doc/uk/ac/ed/inf/utils/Initialised.html]

Initialised


---


|  |  |  |
| --- | --- | --- |
| **Overview** \| **Package** \| **Class** \| **Use** \| **Tree** \| **Deprecated** \| **Index** \| **Help** | |  |
| **PREV CLASS**   **NEXT CLASS** | **FRAMES**    **NO FRAMES**     **All Classes** |  |
| SUMMARY: NESTED \| FIELD \| CONSTR \| METHOD | DETAIL: FIELD \| CONSTR \| METHOD |  |


---


## uk.ac.ed.inf.utils Class Initialised

```
java.lang.Object
  uk.ac.ed.inf.utils.Initialised
```

**All Implemented Interfaces:**: Initialisable

---

``` public class Initialised extends java.lang.Object implements Initialisable ```

---

| **Field Summary** | |
| --- | --- |
| `boolean` | `m_initialisationIsCorrect` |


**Constructor Summary**

`Initialised()`


| **Method Summary** | |
| --- | --- |
| `boolean` | `initialisationIsCorrect()` |
| `void` | `setInitialisation(boolean isCorrect)` |
| `void` | `setInitialisationIsCorrect()` |
| `void` | `setInitialisationNotCorrect()` |

**Methods inherited from class java.lang.Object**

`equals, getClass, hashCode, notify, notifyAll, toString, wait, wait, wait`

**Field Detail**

### m\_initialisationIsCorrect

```
public boolean m_initialisationIsCorrect
```


**Constructor Detail**

### Initialised

```
public Initialised()
```


**Method Detail**

### initialisationIsCorrect

```
public boolean initialisationIsCorrect()
```

:   **Specified by:**: `initialisationIsCorrect` in interface `Initialisable`

---


### setInitialisation

```
public void setInitialisation(boolean isCorrect)
```

:   **Specified by:**: `setInitialisation` in interface `Initialisable`

---


### setInitialisationIsCorrect

```
public void setInitialisationIsCorrect()
```

---


### setInitialisationNotCorrect

```
public void setInitialisationNotCorrect()
```


---


|  |  |  |
| --- | --- | --- |
| **Overview** \| **Package** \| **Class** \| **Use** \| **Tree** \| **Deprecated** \| **Index** \| **Help** | |  |
| **PREV CLASS**   **NEXT CLASS** | **FRAMES**    **NO FRAMES**     **All Classes** |  |
| SUMMARY: NESTED \| FIELD \| CONSTR \| METHOD | DETAIL: FIELD \| CONSTR \| METHOD |  |


---
